# A challenge of improving visibility of the research on Assisted Reproduction in Latin America

**DOI:** 10.5935/1518-0557.20140083

**Published:** 2014

**Authors:** José Gonçalves Franco Junior

**Affiliations:** SBRA President

In 2014, the JBRA-Assisted Reproduction (official publication of the Brazilian Society of Assisted Reproduction, Latin American Network of Assisted Reproduction and Pronúcleo-Brazilian Association of Embryologists) will be an online open-access publication in the English language. The articles may be searched and opened in PDF format at no cost.

The journal has been designated for specialists and researchers in the health sciences, specifically, gynecologists, andrologists, biologists, urologists and embryologists.

Basic and clinical studies in the areas of assisted reproduction, infertility, reproductive genetics, reproductive immunology, andrology, reproductive microbiology, laboratory techniques in assisted reproduction and gynecological endocrinology are welcome.

Today, our database contains 25,000 professionals worldwide who receive online issues of JBRA-Assisted Reproduction. Additionally, the process of indexing in “PubMed” is evolving; however, we need to increase the number of articles without sacrificing quality.

The goal of these modifications is to obtain an appropriate impact factor for JBRA-Assisted Reproduction through the dissemination of the scientific production of Latin America. This mission is not simple because the transition process requires intellectual donation, i.e., preferential submission to JBRA-Assisted Reproduction in lieu of publishing in more established journals. The editors and scientific reviewers of JBRA-Assisted Reproduction are invested and prepared for this transformation, but we also require strong and cohesive support from authors.

Hopefully, the winds blow our way...

**José Gonçalves Franco Junior**

SBRA President

